# Closed-loop wearable naloxone injector system

**DOI:** 10.1038/s41598-021-01990-0

**Published:** 2021-11-22

**Authors:** Justin Chan, Vikram Iyer, Anran Wang, Alexander Lyness, Preetma Kooner, Jacob Sunshine, Shyamnath Gollakota

**Affiliations:** 1 Paul G. Allen School of Computer Science and Engineering, University of Washington, Seattle, WA USA; 2 Department of Electrical and Computer Engineering, University of Washington, Seattle, WA USA; 3 Department of Anesthesiology and Pain Medicine, University of Washington, Seattle, WA USA; 4 West Pharmaceutical Services, Exton, PA USA

**Keywords:** Biomedical engineering, Electrical and electronic engineering, Public health

## Abstract

Overdoses from non-medical use of opioids can lead to hypoxemic/hypercarbic respiratory failure, cardiac arrest, and death when left untreated. Opioid toxicity is readily reversed with naloxone, a competitive antagonist that can restore respiration. However, there remains a critical need for technologies to administer naloxone in the event of unwitnessed overdose events. We report a closed-loop wearable injector system that measures respiration and apneic motion associated with an opioid overdose event using a pair of on-body accelerometers, and administers naloxone subcutaneously upon detection of an apnea. Our proof-of-concept system has been evaluated in two environments: (i) an approved supervised injection facility (SIF) where people self-inject opioids under medical supervision and (ii) a hospital environment where we simulate opioid-induced apneas in healthy participants. In the SIF (*n* = 25), our system identified breathing rate and post-injection respiratory depression accurately when compared to a respiratory belt. In the hospital, our algorithm identified simulated apneic events and successfully injected participants with 1.2 mg of naloxone. Naloxone delivery was verified by intravenous blood draw post-injection for all participants. A closed-loop naloxone injector system has the potential to complement existing evidence-based harm reduction strategies and, in the absence of bystanders, help make opioid toxicity events functionally witnessed and in turn more likely to be successfully resuscitated.

## Introduction

Opioid overdose deaths remain a major public health problem, with mortality increasing during the COVID-19 pandemic^1–3^. A sizable majority of these deaths involve the nonmedical use of opioids, particularly fentanyl^4,5^. Opioid overdose leads to death due to respiratory failure, which is characterized by respiratory depression and sedation, resulting in a hypoxemic/hypercarbic state^6^. Left untreated, this physiologic combination can lead to cardiac arrest, anoxia, and death. A unique feature of opioid toxicity is that it is readily reversed with naloxone, a competitive antagonist which rapidly restores respiration and decreases sedation^7^. Accessibility to naloxone as a harm reduction intervention for people at risk for opioid overdose is a focus of public health interventions to combat the rising number of opioid-related deaths^8^.

Increasing access to naloxone is a necessary component of harm reduction. However, it does not address instances when there is no bystander to administer the antidote or when the event goes unrecognized by a witness. Thus, to complement evidence-based harm reduction strategies (e.g., carrying naloxone, using with others, spaces for supervised injection)^9–12^ there is a critical need for technologies that can potentially make all opioid toxicity events functionally witnessed.

In this work, we develop a proof-of-concept closed-loop, wearable naloxone injector system capable of administering naloxone in the setting of an apnea, specifically prolonged apnea. Building this system requires addressing a number of design and validation challenges. Detecting apnea events require both a sensor that can measure reduced, opioid-induced respiration accurately as well as a processing algorithm that can robustly identify the prolonged apnea events of interest. Moreover, the sensing and processing elements must be low-power to achieve a wearable, battery-powered solution that can operate without user intervention. In addition, clinical validation of the system introduces challenges of its own due to the high-risk nature of non-medical opioid use as well as the practical issues with testing a custom device with human participants.

Our three-part solution comprises a detector element (a pair of on-body accelerometers that measure respiration and an onboard microcontroller that detects apneic motion), a commercially available wearable injection system that administers the drug subcutaneously and an actuator to activate the injector system in the presence of prolonged apnea events. We demonstrate low-power on-device processing to extract breathing rates and detect apneic motion. Our system also has the ability to transmit the data to a nearby smartphone using Bluetooth. To demonstrate proof-of-concept of this system, we conducted testing on participants (Table 1) in two environments: (1) a supervised injection facility in Vancouver, BC, where people self-inject opioids in the presence of medical personnel, and (2) a hospital environment.

**Table 1:** Demographic summary of participants used for algorithmic evaluation of overdose detection and breathing rate tracking

**SIF (*n*** = **25)**	
Age (years)	48 ± 13
Height (cm)	175.2 ± 8.2
Weight (kg)	77.2 ± 21.4
Body mass index	25.0 ± 5.7
Sex	
Male, *n* (%)	22 (88)
Female, *n* (%)	3 (12)
Race	
Asian, *n* (%)	1 (4)
Caucasian, *n* (%)	19 (76)
First Nations, *n* (%)	2 (8)
Persian, *n* (%)	1 (4)
Not recorded, *n* (%)	2 (8)
Drug injected	
Fentanyl, *n* (%)	3 (12)
Heroin, *n* (%)	6 (24)
Unknown, *n* (%)	1 (4)
Not recorded, *n* (%)	17 (68)
**Hospital (*n*** = **20)**	
Age (years)	33 ± 10
Height (cm)	172.2 ± 8.7
Weight (kg)	76.0 ± 22.6
Body mass index	25.6 ± 7.6
Sex	
Male, *n* (%)	9 (45)
Female, *n* (%)	11 (55)
Race	
Asian, *n* (%)	4 (20)
Black, *n* (%)	1 (5)
Caucasian, *n* (%)	14 (70)
Hispanic or Latino, *n* (%)	1 (5)

## Results

### Concept and prototype

**Figure 1:**
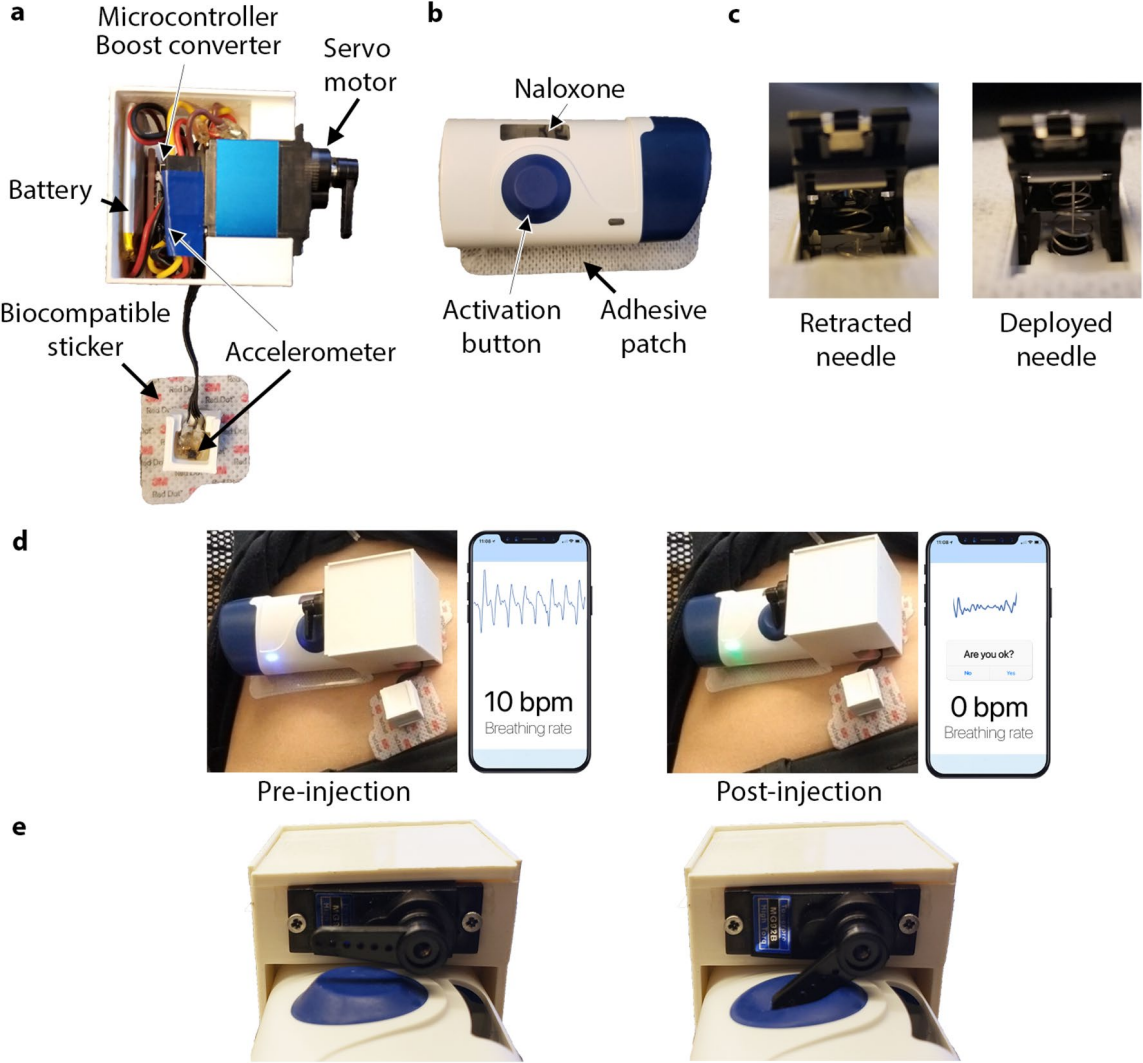
Overview of wearable auto-injector. (**a**) Sensor patch consists of two accelerometers to detect respiration and apneas, as well as a servo motor to activate the injector in the event of an overdose. (**b**) Wearable injector delivers naloxone subcutaneously when activation button is pressed. (**c**) The injector needle in a retracted and deployed state. After injection, the needle-shield locks out over the sharp end of the needle to prevent possible injury. (**d**) The device as placed on the subject’s abdomen prior to and after activation of the injector. (**e**) Close-up view of servo motor pressing the injector button.

Our wearable device consists of an accelerometer-based sensor patch for detecting respiration and motion, and a commercially available wearable injection system (SmartDose$\circledR$ 3.5 Injector, West Pharmaceutical Services, Exton, PA)^13^ (Fig. 1a,b). The commercially available injector system, however, requires the user to manually push a button to deliver the drug and does not have embedded sensors to measure human physiological signals (e.g., breathing). To create a closed-loop system, we create an accelerometer-based sensor patch and an actuator. Accelerometers are commonly used in smartphones, fitness trackers and many other consumer devices to measure gross motion. These sensors are available in small form factor, low cost, and low-power packages. We designed algorithms to measure both gross body motion and respiration patterns using accelerometers when placed on the body. Our system is programmed to use cessation of coarse and breathing motion as indicators of an opioid overdose. In such an event, it will activate the injector and deliver naloxone subcutaneously.

A challenge with using data from a single accelerometer is being able to reliably track diminished respiratory motion, which occurs in the setting of opioid use. An accelerometer measures the sensor’s acceleration relative to the force of gravity. Prior work^14^ has shown that performing a double integration of acceleration values to calculate translational motion magnifies noise and error in individual measurements. This is because accelerometer data in practice is not bias-free, and bias increases at a rate of $t^2$ after double integration of the signal where *t* is the time. There are two limitations to using a single accelerometer design. First, the result is prone to interference such as involuntary body motion, which introduces an additive time-variant bias that hinders correct respiration detection. Second, even though most surfaces on the chest and abdomen rise and fall during inhalation and exhalation, the single accelerometer may be placed in a position on the abdomen that only exhibits a small amount of rotational change, which the accelerometer is not sensitive enough to detect (see Supplementary Fig. 2). Further, we observe that in some subjects, particularly those with a higher body mass index (BMI), it can be challenging to reliably extract the breathing signal using a single accelerometer. Two reasons account for this. First, the abdominal movement in these subjects can be shallower due to increased effort required during respiration. Second, the amount of breathing motion can vary significantly between persons based on where the sensor is placed on their abdomen. To address this, we used two accelerometers to detect rotation at two points of the abdomen, with each separated by a small distance. This reduces the likelihood that both the two positions suffer from minimized rotational change. Additionally, this allows us to combine the signals across the two accelerometers to more accurately extract breathing motion.

Our sensor patch consists of two three-axis accelerometers (Bosch Sensortec BMA400) placed on the right lower or left lower quadrant of the subject’s abdomen (Supplementary Fig. 2). The first is attached rigidly to the injector, and the second is placed approximately 3 cm above or below it and is connected using a flexible silicone linkage. This flexible connector allows both accelerometers to rotate independently of each other, which results in more accurate and robust measurements of motion and respiration. The device’s accelerometers are connected to an onboard microcontroller (Nordic Semiconductor nRF52840) and housed in compact 3D-printed cases as shown in Fig. 1. The electronics are powered by a lithium polymer (LiPo) battery. The devices are attached to subjects using the adhesives from two EKG electrodes (3M 2670-5) as well as the adhesive on the back of each single-use injector.

The microcontroller (Nordic Semiconductor nRF52840) runs a motion detection algorithm in real-time to track the subject’s body motion and respiration over time. Cessation of breathing is detected if the algorithm detects a complete cessation of body movements and respiration longer than a programmable threshold. In our hospital study evaluating the device on healthy participants simulating opioid-induced apneas, we set the threshold to 15 s for a proof of concept, as asking participants to cease respiration for longer could cause discomfort. However, alternative respiration thresholds such as a longer apneic time or a sequence of repeated apneas could also be used. We note that while the American Academy of Sleep Medicine defines apneas lasting for as short as 10 s^15^, using such a low threshold is likely to result in false positives in an opioid use context. Indeed, in our prior work^16^, we observed that opioid overdose events requiring resuscitation were often preceded by post-injection central apneas lasting $\ge$ 30 s or a sequence of repeated central apneas, yet several people experienced isolated apneic events (~10 seconds) without experiencing an overdose. This is in agreement with other prior work which has shown that multiple apneas can be a precursor to an opioid overdose^17,18^. The United States Food and Drug Administration (FDA) defines an apnea event as cessation of breathing for 10 s^15^, a threshold in the high-risk opioid use case that would likely be unacceptably low. While using this threshold demonstrates proof of concept, longer durations could be used to avoid false positives in the event of isolated apnea events. Additionally, to provide additional contextual information, a phone-based alert system could be used to determine a person’s state of consciousness in the event of an apnea, and only activate the injector if, for example, the person does not respond to a prompt or other audible alert.

The current system, upon detecting an apneic event, activates the injector (SmartDose$\circledR$ 3.5 Injector). The commercially available version of the injector was designed to have a person trigger an injection by pressing a button; we automate this process by using a servo motor (Towerpro MG92B) to depress the activation button on the device. The injector then deploys a 29 gauge needle into the subject’s skin and delivers the drug to the subcutaneous region (Fig. 1d,e). We note that while this prototype uses the commercially available version of the injector to demonstrate a proof of concept, future versions could be further optimized to reduce form factor. For example, the electronics could be integrated into the injector housing itself.

Prior work^16,19,20^ in this domain has converted a smartphone into a short-range active sonar to contactlessly detect respiratory depression and apneas which are precursors to an opioid overdose. In this study, because participants already have an injector on their body, integrating the overdose detection hardware into a single wearable system reduces the need for multiple electronic devices. Leveraging the active sonar technique would require an additional smartphone device to perform the overdose detection. Prior work has also combined accelerometer data with electrodermal activity, skin temperature to detect opioid intake^21^ as well as identify recurrent opioid toxicity after the effects of naloxone have worn off^22^. Our work differs as we build a closed-loop system which can deliver a single dose of naloxone immediately following respiratory physiology consistent with an opioid overdose.

### Real world opioid use events

**Figure 2:**
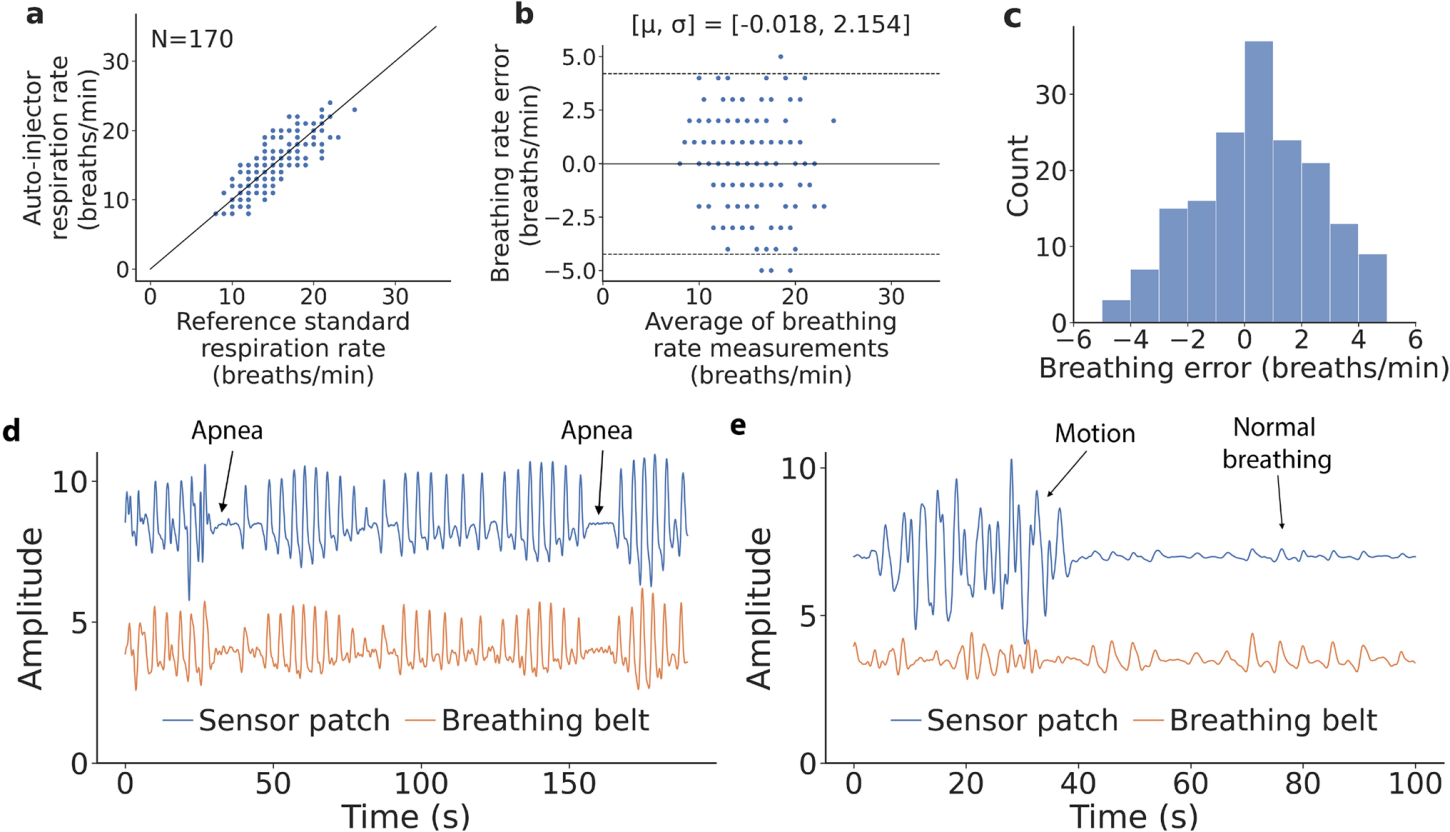
Measurement of opioid use events in the supervised injection facility. (**a**) Correlation and (**b**) Bland–Altman plots comparing breathing rate from respiration belt and wearable sensor patch. In the Bland–Altman plot $\upmu$ is the mean error and $\upsigma$ is the standard deviation (SD) of the errors, the solid line represents the mean error and the dotted lines represent the 95% limits of agreement. (**c**) Histogram of breathing rate error. (**d**) Breathing signal from sensor patch and breathing belt showing normal breathing, post-injection apneas and (**e**) human motion events at the supervised injection facility.

In this study, we evaluated our wearable system at a supervised injection facility in Vancouver, BC. People who use this facility self-inject opioids in the presence of trained personnel who can resuscitate them in the event of an overdose. The goal of this study was to evaluate if the sensor component of the system was able to accurately track respiration in a real-world opioid use environment. Additionally, we measured if real-world non-medical opioid-induced apneas could be detected by our system, given that this type of breathing is a precursor to a potentially fatal overdose event. By design, no naloxone from the prototype was administered to individuals within the supervised injection facility, given the risks associated with un-indicated naloxone administration, which can precipitate withdrawal. There were no adverse reactions to the device across all subjects in the study.

We recruited 25 participants over the course of two visits with a mean age of 48 ± 13 years, and female-to-male ratio of 0.14 (Table 1). Of the 8 (32%) participants that reported the type of opioids being used, 3 (12%) reported using fentanyl, 6 (24%) reported using heroin, and 1 (4%) reported use of an unknown opioid. Two (8%) reported using multiple opioids. After providing informed consent, participants were fitted with our system and a breathing belt that provided reference measurements of respiration rate. Once fitted with the equipment, participants would prepare their opioids and self-inject them, after which their respiration was captured for up to 5 min. Bland–Altman analysis (Fig. 2a,b) showed a bias error of − 0.018 breaths per minute with 166 of 170 samples falling within the 95% agreement limits. The mean absolute breathing rate error computed over 30-s epochs was 1.7 ± 1.3 breaths per minute across all the participants (Fig. 2c). After injection of non-medically indicated opioids, 2 (8%) participants experienced at least one apnea event. In these participants, a decrease in respiratory rate of 3–4 breaths per minute during the monitored post-injection period was detected by our algorithm. Our algorithm did not detect a similar decrease in the respiratory rate of other subjects. During the study, none of the participants required manual intervention or experienced an overdose event.

Figure 2d shows the respiratory pattern obtained from the wearable sensor patch and the breathing belt for a participant after injection. The figure shows that when the subject is stationary we can detect periods of regular breathing as well as identify opioid-induced apneas. In the SIF, many participants would move their body freely throughout a measurement, often waving their arms or turning in their chair. Figure 2e shows high amplitude body motion caused by the subject moving their arms followed by normal respiratory motion. These large motions patterns overpower the subdued breathing patterns detectable by the sensor patch. While the noise from this motion might typically be considered undesirable, it can also serve as a surrogate indicator that a person may not be overdosed. Thus, our system does not need to identify respiration rate continuously in the presence of coarse body motion for the use case of overdose detection. Across all participants, 53.8% of 30-s epochs were considered as breathing epochs, and the remaining were considered as motion epochs.

### Simulated overdose detection

**Figure 3:**
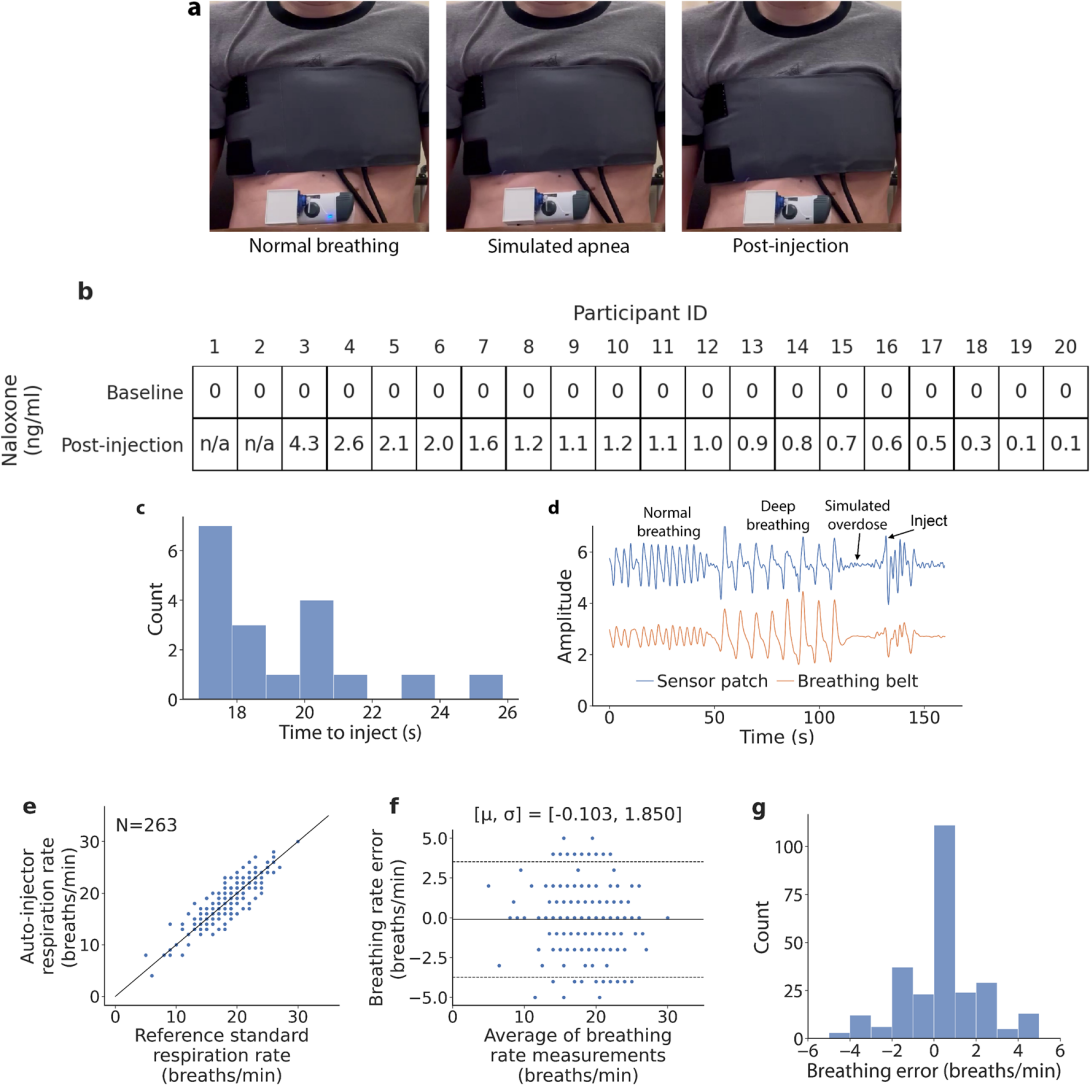
Naloxone delivery in simulated laboratory environment. (**a**) Depiction of participant in laboratory with wearable device and breathing belt. Participant engages in normal breathing, followed by a simulated apnea which activates the injector. (**b**) Naloxone levels in participant blood samples before and after injection. (**c**) Histogram of the time taken for the sensor patch to detect the onset of a simulated overdose and activate the injector. (**d**) Breathing signal from sensor patch and breathing belt showing a simulated apnea and activation of the injector. (**e**) Correlation and (**f**) Bland–Altman plots comparing breathing rate from respiration belt and wearable sensor patch. (**g**) Histogram of breathing rate error.

On account that we were unable (from an ethical standpoint) to administer naloxone with the prototype in the SIF, we sought to validate the preliminary efficacy of our system end-to-end with simulated overdoses in the hospital laboratory setting. In this study, subjects were first fitted with a respiratory belt around their chest to obtain a reference measure of respiration. Our wearable device consisting of a sensor patch and injector was then attached to the left lower quadrant of their abdomen (Supplementary Fig. 2) as per the instructions for use for the SmartDose$\circledR$ 3.5. We simulated overdose events in healthy participants by first having them breathe normally to obtain a baseline measure of breathing before simulating apnea through a 15 s breath-hold (Fig. 3a). This procedure is intended to mimic the cessation of respiration that occurs in the case of an actual opioid overdose. When the wearable system detects that the subject has not moved for at least 15 s it would activate and begin injecting 1.2 mg (0.4 mg/ml, 3 ml total volume) of naloxone into the participant, which is a subcutaneous dosage that is safe for a human volunteer and within the range of an initial dose that someone experiencing an overdose may receive^23–25^. To detect the level of naloxone in the participant’s blood stream, 10 ml of blood was drawn through an intravenous line before and after the injection. Blood samples were subsequently analyzed using mass spectrometry (see Methods) for the presence of naloxone. There were no adverse reactions to the device, injection or naloxone across all subjects in the study.

The IRB for the study was approved for recruitment of 20 participants. The first two initially recruited participants, once apnea was detected, it was observed that the injector did not reliably penetrate the skin and deliver the full dose of naloxone. While the apnea was accurately classified, this likely occurred due to not being adequately coupled to the skin. For the subsequent 18 participants, the injector was adhered to firm and flat areas of the skin on the left lower quadrant of the abdomen, as per the manufacturer’s instructions for use, resulting in a more reliable coupling.

For these 18 participants, the mean age was 32 ± 10 years, and female-to-male ratio of 1.6 (Table 1). Our algorithm correctly identified the cessation of coarse motion and breathing for all participants and successfully activated the injector, without any false activations. Fifteen out of 18 participants triggered the injector on their first breath hold, while 3 of the remaining participants took between 2 to 6 tries due to involuntary movements during the simulated breath hold. It took between 16.9 to 25.9 s from the breath hold for the injector to activate with a mean of 19.4 ± 2.3 s (Fig. 3c). Subgroup analysis reveals that the average time for the injector to activate for the 11 female and 7 male participants was 19.0 ± 1.9 and 20.2 ± 2.9 s respectively. For the 12 participants with body mass index (BMI) < 25 (underweight and normal weight) and the 6 participants with BMI $\ge$ 25 (overweight and obese) the average time to injection was 19.8 ± 2.5 and 18.6 ± 1.8 s respectively. These results suggest that sex and BMI did not have a noticeable effect on our system’s ability to detect a simulated overdose.

Naloxone was detected in all blood samples after injection with the dose amount ranging from 0.1 to 4.3 ng/ml with a mean of 1.2 ± 1.1 ng/ml (Fig. 3b). Baseline blood samples collected prior to injection contained no naloxone. On subgroup analysis we found that the amount of naloxone detected in female and male participants was 1.1 ± 1.3 and 1.3 ± 0.7 ng/ml respectively. For participants with BMI < 25 and $\ge$ 25, the amount of naloxone in the blood samples was 1.2 ± 1.2 and 1.1 ± 0.6 ng/ml, respectively. These findings do not show substantial differences in naloxone absorption by sex or BMI.

Figure 3d shows the respiratory waveform obtained from the breathing belt and the sensor patch for the subject performing breathing exercises, simulating an apnea and being injected with naloxone in the laboratory. Breathing rate was not computed for two participants due to a data collection error with the breathing belt. Across all 263 30-s breathing epochs obtained during the study, the inter-class coefficient was $R=0.893$ (Fig. 3e). On Bland-Altman analysis the bias error was − 0.103 breaths per minute with 28 out of 263 points falling out of 95% agreement limits (Fig. 3f). The mean absolute breathing error was 1.3 ± 1.4 breaths per minute (Fig. 3g). Breathing rate accuracy for participants in the hospital laboratory was higher than those in the SIF as participants were more compliant and sat still in a chair for the duration of the study. Additionally, we found that the injection and blood draw process caused participants to be more conscious of their breathing, which resulted in taking deeper breaths.

### Subgroup analysis of breathing accuracy

**Figure 4:**
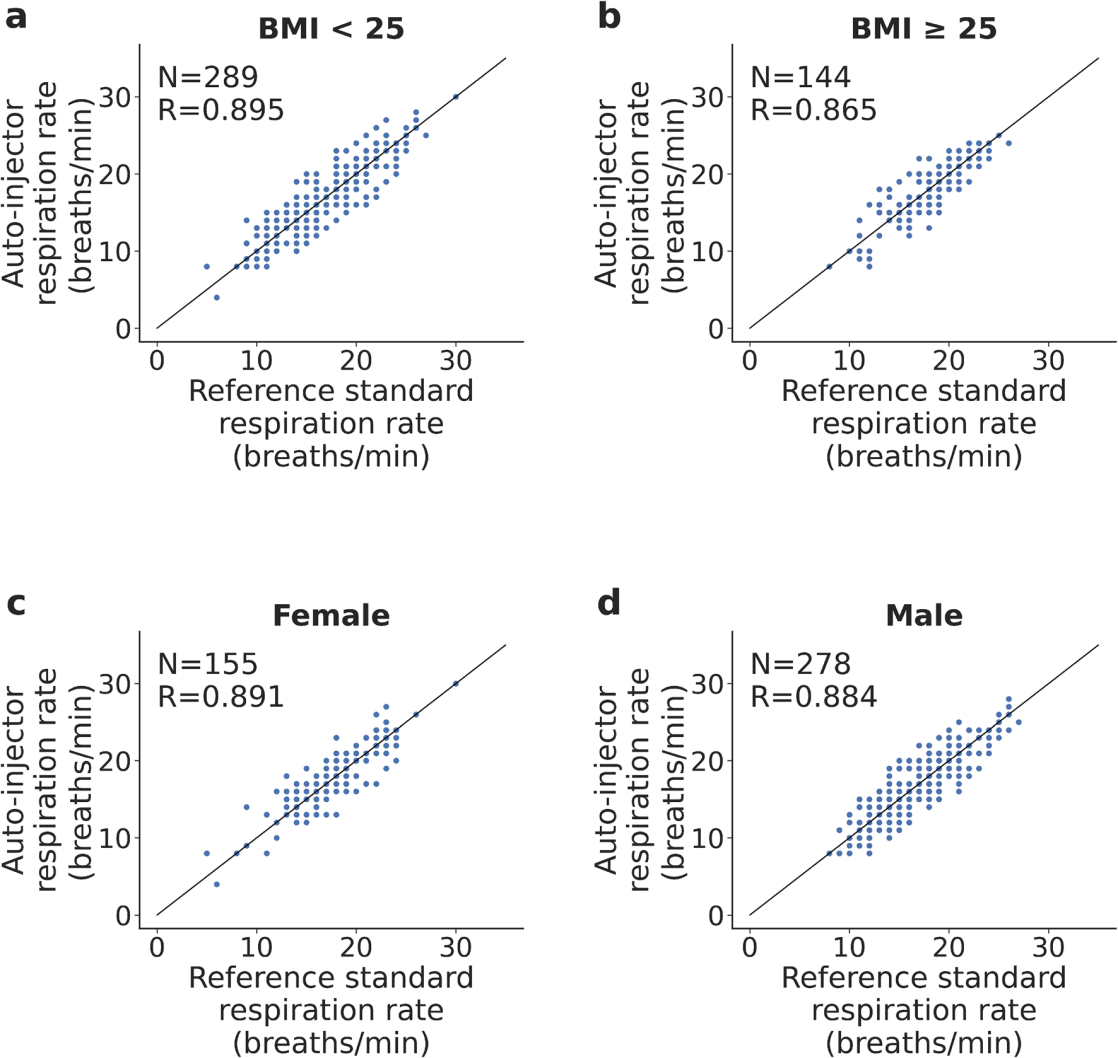
Subgroup analysis of breathing rate accuracy across participants from the SIF and the simulated laboratory setting. (**a**–**b**) Correlation plots comparing breathing rate from respiration belt and wearable sensor patch for participants with BMI < 25 (underweight and normal weight), BMI $\ge$ 25 (overweight and obese), and (**c**–**d**) female and male participants.

We examine the effect of BMI and sex across all breathing epochs collected from the SIF and the hospital environment (Fig. 4a–d). Data is pooled across both studies to maximize the number of breathing epochs in each demographic group. Subgroup analysis for BMI shows that the inter-class correlation coefficient for BMI < 25 and BMI $\ge$ 25 participants was $R=0.895$ and $R=0.865$ respectively, which suggests the error in the breathing rate was slightly higher in overweight and obese participants. The mean absolute breathing rate error for the two BMI classes is 1.4 ± 1.4 and 1.5 ± 1.3 respectively. The inter-class correlation coefficient for female and male participants is $R=0.891$ and $R=0.884$ respectively, while the mean absolute breathing rate error is 1.3 ± 1.4 and 1.5 ± 1.4 respectively. These findings suggest that tracking breathing rate was slightly more accurate in female participants, but the difference is not statistically significant.

## Discussion

Fatal drug overdoses in the United States are at an all time high, with over 81,000 such deaths recorded between May 2020–2021^26^, and 70,630 such deaths recorded in 2019, prior to the COVID-19 pandemic^27^. Of these deaths, opioid overdoses account for the majority, responsible for 70.6% of all fatal drug overdoses in 2019^27^. Opioid overdoses can be reversed rapidly with naloxone, a drug that is widely available to emergency medical services (EMS) and increasingly the general public. However, up to 51.8% of fatal overdoses occur when the person is alone, and in 27.4% of cases bystanders do not recognize the symptoms of an overdose and are not able to react in a timely manner^28^. Additionally, EMS may not always be able to respond promptly if the overdosed patient is in a less accessible location, such as in rural areas. Here, we present a closed-loop sensing system that is able to accurately measure real world opioid-induced breathing events and deliver naloxone in a timely manner.

There is no consensus on the ideal naloxone dosage to overcome an acute opioid overdose however guidelines recommend starting dosages of 0.04 to 0.4 mg for intravenous and intramuscular delivery to avoid provoking opioid withdrawal in patients, with repeated doses up to 2 mg recommended until the subject’s respiration returns to normal^29,30^. Prior studies delivering naloxone subcutaneously began with a starting dosage of 0.8 mg of naloxone with a subsequent dose of 0.8 mg if needed^23,24^. We note that inter-individual variation in the rate of naloxone absorption can be attributed to the interplay between physiological factors such as subcutaneous blood flow, metabolism, skin temperature, obesity, blood glucose levels, body position and whether the subject smokes or exercises^31,32^. As such, testing on healthy, non-overdosed volunteers could lead to naloxone blood level that may not be representative of the intended use population. This threat to external validity is mitigated by other real-world evidence demonstrating comparable recovery intervals of opioid overdose victims receiving subcutaneous vs. intravenous naloxone^23^. Nevertheless, because this system utilizes a device, further testing would be warranted to test subcutaneous absorption in the population of interest (i.e., those experiencing an opioid overdose).

A review^33^ of different routes of naloxone administration showed that the bioavailability of naloxone via intramuscular and subcutaneous routes was 98.3%, and via the intranasal route was 43.9–51.9%. Prior work^23^ showed that there was no clinical difference in time to reverse an opioid overdose when comparing 0.8 mg of naloxone delivered subcutaneously against 0.4 mg delivered intravenously, due to the additional time required to establish an intravenous line. In a double-blind, randomized clinical trial comparing the effects of the same dosage of naloxone, participants were less likely to require a rescue dose of naloxone after intramuscular administration, compared to intranasal administration^34^.

Our study has several limitations. Usability and avoidance of stigma are crucial considerations when designing a harm reduction system for nonmedical opioid use. Subsequent human factor studies are needed to assess the comfort and discreetness of the device when worn for longer periods of time, particularly in unsupervised settings. In a recent study^28^ involving 97 adults with an opioid-use history of at least three months 76% of participants were willing to wear a closed-loop device for sensing an overdose and delivering a reversal agent upon detection. Almost all (75.5%) respondents indicated a willingness to wear the device all or most of the time.

It is beyond the scope of this current study to evaluate the device in real world nonmedical opioid use situations. Due to safety concerns, only healthy participants were recruited and tested for simulated overdoses in a laboratory environment under the supervision of an anesthesiologist. The ability of our device to accurately track respiratory change amongst people who use opioids have been validated in the SIF. We note that a relatively small proportion of opioid users in the SIF (2 out of 25 participants) experienced apnea events. Future studies to monitor more participants over a longer period of time would be needed to evaluate the device’s apnea detection algorithm on real world participants at risk for an overdose event when they are using.

Future studies are needed to evaluate end-to-end testing of naloxone injection with our device in people who use opioids for non-medical purposes. Additionally, while the sensor analyzes chest and abdominal motion (i.e., it does not use photoplethysmography for measurements through the skin) future studies should test across a more racially heterogeneous population to assess generalizability of the device. In terms of comfort, further optimization could be beneficial to reduce the device’s form factor. A longitudinal study could also assess the long-term wearability, discreetness and comfort of the device over time. In this work, a research assistant attached the device to all participants. Subsequent usability studies are necessary to assess if people are able to successfully place the device on their body and detach it on their own.

Accelerometer-based systems to detect respiration have been applied to the detection of different breathing patterns^35^. Multiple accelerometer designs with two sensors to measure respiration^36^ and up to ten sensors^37^ have been designed to investigate optimal placement for respiratory measurements. Other methods of measuring respiration include mechanical sensors based on thin film^38^, cotton^39^ and graphene^40^ to measure the strain and capacitance of torso movement that are cotton-based; microphones placed on the throat to listen to airflow^41^. Radar, optical and thermal approaches have also used to contactlessly measure respiration rate^42^. Our system builds on prior work by using a dual-accelerometer design for the specific purpose of detecting opioid-induced apneas. Our design is unique in that it has been evaluated on people who engage in high-risk opioid use activities within a supervised injection facility. Unlike prior systems, our sensor design is also part of a closed-loop system that has been evaluated to detect overdoses in real-time and perform drug delivery, with confirmed naloxone presence in the blood.

Recent closed-loop implanted systems^43^ for delivery of naloxone propose the use of an EKG sensor for respiratory monitoring and a high frequency magnetic field to heat an implant to $42^{\circ }\mathrm{C}$ and melt a capsule containing naloxone. The system has been tested in mice and requires further testing to reduce issues with naloxone leakage. In contrast, our wearable system does not require any heating elements or surgical implantation, which reduces safety concerns, and has been tested end-to-end on human participants. We mitigate concerns about the safety and reliability of the injector by leveraging an existing commercially available injector which has been extensively tested as a drug delivery platform.

As fatal drug overdoses continue to increase, new strategies are required to reduce mortality and morbidity rates. While existing harm reduction interventions like take-home naloxone programs and supervised injection facilities have been efficacious, there is a critical need for systems that can reverse opioid toxicity events especially in the absence of bystanders, where many overdoses occur. In this work, we develop a closed-loop naloxone injector system that can identify real-world and simulated opioid-induced apneas and deliver naloxone subcutaneously. Such a system has the potential to readily reverse overdoses particularly when the event is unwitnessed. A closed-loop system is useful even in scenarios when overdose events are witnessed as neither friends nor family may be aware that an overdose is occurring. Our proof-of-concept system can also be used to alert EMS to people experiencing an overdose, who can then provide additional care for patients and help ensure that breakthrough opioid toxicity does not lead to harm. Such a system complements current methods of evidence-based harm reduction and could help ensure that more overdoses that would otherwise be unseen and un-resuscitated could become functionally witnessed and reversed.

## Materials and methods

All participants provided informed consent and the studies were approved by the University of Washington Institutional Review Board (STUDY00005944), and was retrospectively registered at ClinicalTrials.gov (Trial-ID: NCT05099614) on 29/10/2021. All studies complied with relevant ethical regulations. Randomization was not applicable and investigators were not blinded.

### Hardware design

Our hardware has two main components: a sensor patch and the injector system.

#### Sensor patch design

We use two accelerometers (Bosch Sensortec BMA400) and connect them to a microcontroller (nRF52840) which processes the data and can stream it over Bluetooth to a companion Android smartphone app for storage and data logging. Data transmission to the smartphone is optional and the wearable injector with our sensor is a self-contained system. The accelerometers, microcontroller and servo motor are powered using a 500-mAh lithium polymer battery ($35.3 \times 29.2 \times 4.9$ mm). A fully charged battery was able to power the system, run the motion detection algorithms, and stream accelerometer data using the microcontroller’s integrated Bluetooth radio to the companion smartphone app over the course of a single study day, which lasted around 7 hours.

To increase the output torque of the servo motor and ensure that it can robustly trigger the injector’s activation button, we use a boost converter circuit (SparkFun PRT-14411) to operate the motor at 5 V. The accelerometers sampled at a rate of 17 Hz which we find is the maximum rate at which we can stream the data to a smartphone and run the motion detection algorithm simultaneously. Accelerometer data is streamed and saved to the smartphone for data logging and analytics.

We use polylactic acid (PLA) plastic as the material for the 3D printed housing, with a wall thickness of 1 mm. The housing that contains the first accelerometer, microcontroller, boost converter, battery and motor has dimensions of $41.9 \times 44 \times 38.6$ mm. The housing containing the second accelerometer has dimensions of $14.5 \times 14.3 \times 17.3$ mm. The flexible linkage connecting both housings has a length of 27.8 mm. The total weight of the sensor patch is 58 g. At the base of both accelerometer housings is a metal button snap connector. During each test, fresh bio-compatible stickers (3M Red Dot Electrodes) are snapped onto these connectors, and the adhesive of the stickers are used to couple the device to the participant’s abdomen.

#### Injector system

Our system is integrated with the SmartDose$\circledR$ 3.5 injector from West Pharmaceutical Services (Exton, PA). The device holds a cartridge which can contain up to 3.5 ml of injectant. For this study 3 ml of 0.4 mg/ml naloxone (1.2 mg total) and 0.5 ml saline was used. The device also has an adhesive patch which is used to adhere to a person’s skin. The front of the device also includes an indicator light that provides feedback before, during, and after use. The injector is activated by a simple button push, which deploys a 29-gauge thin wall needle subcutaneously to begin the drug delivery process. At this point, the indicator light blinks green to indicate that the ongoing process is running. The device uses its internal motor to deliver the full 3.5 ml dose from the cartridge, in our case, 1.2 mg of naloxone (Hospira) and 0.5 ml saline, over the course of 9 min, at a dose rate that was pre-programmed for this particular model. The wearable injection device from West is separately powered from the sensor patch by three 1.55 V button cell batteries.

We note that the SmartDose$\circledR$ 3.5 is FDA approved for use in combination with the drug Repatha (Amgen) and is thus pre-programmed for delivery over 9 min^44^. The device can in principle be pre-programmed to deliver drug at a faster rate^45^. While this prototype uses a version of the SmartDose$\circledR$ approved for human use to demonstrate a proof of concept and evaluate it with human participants, future versions would be programmable to inject naloxone more rapidly, given the emergent nature of opioid overdose. Finally, the form factor of the system can be reduced by integrating the electronics into the injector housing itself.

### Algorithm to identify breathing cessation

We use the accelerometer data from the sensor patch to identify breathing cessation that then triggers the injector’s activation button (Supplementary Algorithm 1). Every 5 s, we calculate the amount of rotational change that occurred on each accelerometer axis in real-time on the microcontroller. If the algorithm detected the cessation of both coarse and fine motion on all six axes on the accelerometer for three 5-s epochs, it would detect an apnea has occurred. The short epoch length of 5 s was selected because we wanted to minimize the amount of time it takes for the algorithm to detect the cessation of breathing event. Note that we use lack of motion for three 5-s epochs to detect an apnea since in the hospital study, participants were asked to hold their breath for 15 s. This can be increased to 30 s to support real-world opioid overdose events^16^.

To determine cessation of movement after a single epoch, the algorithm first iterates through each axis and sums the absolute amplitude changes on that axis, as a measure of the amount of motion. If the motion for a given axis is below an amplitude threshold $trigger\_thresh$, that indicates there was a cessation of movement on that axis. If a cessation of motion is observed for three consecutive 5-s epochs for all six axes, our algorithm then notes that an apnea has occurred. At this point in the study, the algorithm also activated the injector to deliver naloxone into the subject. In our implementation, $trigger\_thresh$ was set to 300.

If any motion above $trigger\_thresh$ is observed on any of the six axes, we reset the trigger counter for all axes. This is a conservative condition used to eliminate false positives and ensure that only a complete cessation of both coarse and fine (e.g., breathing) motion will result in an apnea detection. No false positive injections occurred during the course of the hospital study.

### Algorithm to compute breathing rate

We compute breathing rate from the accelerometer signals using three key steps (Supplementary Algorithm 3). First, we apply a filter on the accelerometer signals to remove high frequency noise and direct current (DC) bias. Second, to increase the signal strength of the target signal, we identify the axis with the strongest signal strength from each accelerometer and sum them. Finally, the zero-crossings of the signal are analyzed using minimum distance and amplitude heuristics to compute breathing rate.

#### Filtering high-frequency bias and DC noise

In this step we filter out high frequency noise greater than 1 Hz and DC bias from the accelerometer signal using a band-pass filter with passband [0.1, 1] Hz. The resultant signal is limited to movement occurring 6 to 60 times a minute, which includes respiratory motion. The band-pass filter is constructed by subtracting the low-pass filtered signal with frequencies [0, 1] Hz from the low-pass filtered signal *DC* with frequencies [0, 0.1] Hz. We use a sinc filter which is an idealized low-pass filter for this step. The filter is applied to the *x*, *y*, *z* axes of both accelerometers.

#### Combining accelerometer signals

The goal of this step is to increase the signal strength of the breathing signal by identifying the strongest axis from each accelerometer, and adding the two axes together (Supplementary Algorithm 2). This procedure involves three steps:

*Step 1: Orienting sensor axis.* We first orient an axis such that inhalation is seen as an increasing signal amplitude while exhalation is seen as a decreasing amplitude. We observe that breathing peaks caused by inhalation are larger in magnitude and prominence than exhalation. Based on this observation we check if the magnitude and prominence of maximums is greater than those of its minimums during a period of baseline breathing. If the maximums are larger, the axis is correctly oriented, otherwise, we negate the signal in order to orient it correctly.

*Step 2: Selecting sensor axes.* Next, we select the axis with highest signal strength from each of the two accelerometers. We define signal strength for an axis as the difference between the highest inhalation peak and lowest exhalation peak. If the signal strength for an axis is below a threshold $signal\_thresh$, no axis is selected for that accelerometer. $signal\_thresh$ was selected as the minimum signal strength under which the signal appeared too close to the noise floor. In our implementation, we set $signal\_thresh$ to 5.

*Step 3: Adding axes across accelerometers.* The final step is to add the axis on the two accelerometers that are identified in the previous step. This addition produces a single stream with a higher signal strength than any one of the accelerometer signals since they were oriented in the same way in Step 1. This combined breathing signal is then used to compute breathing rate below.

#### Computing breathing rate

Finally, we compute the breathing rate using this processed signal. As the DC bias in the filtered signal is removed, it is centered around zero. As a result of this circumstance, we can identify the zero-crossings to determine when the subject has inhaled or exhaled. Specifically, two zero-crossings represents a single inhalation and exhalation cycle, and corresponds to a single breath. In our study, we compute breathing rate in 30-s epochs for each participant. However, noise can cause the signal to fluctuate around zero, resulting in zero-crossings that are not indicative of breathing. To reduce these false crossings we apply two heuristics. In the first heuristic, we set a threshold for the minimum amount of time that should occur between zero-crossings. We set the sample threshold to be 350 ms. In the second heuristic, we set a threshold for the minimum change in amplitude that occurs between zero-crossings. We select an amplitude threshold which rejects the zero-crossings caused by noise. We find that per-participant thresholds do not improve breathing rate accuracy, as a participant’s breathing pattern can change in speed and amplitude at different points in a measurement. So, we use the same predetermined threshold value of 60 for all participants which rejects zero-crossings caused by noise.

### Distinguishing coarse motion from breathing

The accelerometer signal is highly sensitive to different coarse motion patterns such as the subject changing sitting positions or rotating their body and limbs. These motion patterns are higher in amplitude and can overwhelm any respiratory motion which is comparatively lower in amplitude. However, in this particular use case involving high-risk opioid use, signs of motion can generally be indicative that a person is still conscious and has not overdosed. Thus, for this specific application, it is less critical to identify the breathing rate in the presence of coarse motion, which can provide insight into the safety status of a person engaging in high-risk opioid use. Nevertheless, we would like to identify which portions of the accelerometer signal contain coarse motion patterns and distinguish them from segments that contain breathing motion. The amount of motion in each 30-s epoch is captured by looking at the variance in the high frequency component of the accelerometer signal that is greater than 1 Hz. The high-frequency signal is segmented into 1 s epochs, and the variance of each segment is calculated to produce a signal *var*. We calculate the following two thresholds: $v_{max}=max(vars)$ and $v_{sum}=\sum vars$. These provide a measure of the amount of motion in each epoch. If $v_{max}$ or $v_{sum}$ is above predetermined thresholds of 50000 and 20000 respectively for a particular epoch, that epoch is classified as being coarse motion.

### Supervised injection facility study

Data was collected in two sessions from consenting people who use opioids at the InSite Supervised Injection Facility (SIF) in Vancouver, BC. The study inclusion criteria were people who injected opioids and use the SIF, over 18 years old, and had capacity to provide informed consent (as determined by InSite staff). The InSite staff identified potential participants at the time they checked into the SIF for the purposes of supervised opioid self-injection. A research assistant then approached them for informed consent. Participants were approached consecutively after check-in into the facility. Upon completion participants received a $5 Starbucks gift card as compensation.

Participants that provided verbal informed consent were, per SIF policy, provided with sterile injecting equipment and directed towards a monitored injection stall. All participants were subject to standard clinical monitoring protocols by the clinical staff present in the SIF. During measurements, clinical staff at the SIF would visually observe participants for signs of an overdose, per standard protocols. During the study, none of the participants experienced an overdose event requiring resuscitation.

Participants were fitted with a respiration monitor (Vernier respiration belt) across their chest with a sampling rate of 10 Hz to obtain a reference standard of respiratory activity. Our sensor device was then placed on the right lower or left lower quadrant of the subject’s abdomen and data was transmitted to a laptop. Subjects were asked to perform injections as they normally would, and were able to move freely.

To observe their baseline breathing, participants were asked to breathe normally for 2 min, after which they were allowed to perform their injections. Subjects were then monitored for 5 min after injection. The period of 5 min was selected as that is a reasonable and high-risk time period during which an opioid overdose would occur, particularly if the opioid is fentanyl. Indeed, after 3–5 min fentanyl reaches peak plasma concentration, and after 5 min 80% of the injectant would have left the plasma^46^. As we note in the main text, two participants experienced post-injection apneas without having an overdose event. Our algorithm correctly detected a decrease in post-injection respiration rate for these participants, which were caused by the apnea events.

### Hospital study with healthy participants

Healthy participants above the age of 18 without an allergy to naloxone or ingredients in its formulation were deemed eligible for the study. Exclusionary criteria include a history of alcohol or substance abuse, kidney failure or liver disease, unusual pain sensitivity or lack of sensitivity, chronic myofascial, inflammatory, neuropathic pain, use of medication known to interfere with naloxone, currently on opioids and pregnant women and nursing mothers. Participants were recruited through a study advertisement circulated throughout our institution, community and by word of mouth. Participants were screened via inclusion and exclusion criteria prior to the study. Female participants underwent a urine pregnancy test to exclude pregnancy at the time of the study. Participants were compensated with a $100 Amazon gift card at the conclusion of their study session.

Participants were fitted with a Vernier respiratory belt across their chest with a sampling rate of 10 Hz to obtain a reference standard of respiratory activity. An attending anesthesiologist then drew a baseline measurement of 10 ml of blood from the participant’s arm via an intravenous line catheter. The intravenous catheter remained in the participant’s arm for the duration of the study.

After the device was attached, a research assistant used a companion smartphone app to send a Bluetooth command to the injector which would start the overdose detection algorithm which ran in real-time on the sensor patch. The research assistant then confirmed the device’s functionality by checking that the accelerometer data was being streamed from the wearable device to the smartphone, and that the algorithm was appropriately detecting motion from the participant.

The participant was then asked to engage in two baseline breathing exercises. In the first exercise, they were asked to breath normally for 1  m. In the second exercise, they were asked to inhale and exhale at the sound of a tone that would sound eight times in a minute, for a duration of 1  min. Participants were then instructed to hold their breath for a period of 20 s to simulate the cessation of breathing that occurs during an opioid overdose. Some participants elected to exhale deeply prior to holding their breath. Additionally, participants were instructed to minimize motion during the 20 s simulated apnea. When the algorithm detected 15 s of inactivity, it would activate the servo motor of the device to press the button on the injector. The injector would then deploy a 29 gauge thin wall needle into the patient’s abdomen and begin administering the 3.5 ml (1.2 mg) of prepared naloxone subcutaneously. Post-injection the attending anesthesiologist drew 10 ml of blood through the intravenous line at 3 and 8 min post-injection.

When the naloxone was fully administered, the intravenous line from the patient’s arm was removed. The vernier respiratory belt and wearable injector were removed thereafter. The biocompatible leads from the injector are disposed, and the device is wiped clean using anti-microbial disinfectant. Patients were monitored for 30 min post-injection to observe for adverse reactions to naloxone.

Blood samples were analyzed at the University of Washington Medicinal Chemistry lab where they underewnt mass spectrometry. The samples were analyzed using the Xevo-TQs mass spectrometer and a Agilent Zorbax-Aq column. The mobile phase was 0.1% formic acid in H_2_O and 0.1% formic acid in methanol. To prepare the samples 10 µl was first added to 20 µl of plasma and mixed in a vortexer. Then 100 µl of MeOH (methanol) was mixed in with a vortexer, and the solution is centrifuged. Supernatant (50 µl) was then mixed with 50 µl H_2_O in an autosampler vial. 2 µl of this solution was then deposited on the Agilent Zorbax-Aq column.

### Statistical analysis

Pearson’s correlation coefficient was computed using inter-class class correlation for correlation plots. The mean bias error and 95% limits of agreement (LOA) were computed for the Bland-Altman plots. LOA was computed as 1.96 times the standard deviation of the error. Statistical analysis was performed using MATLAB, and the figures were generated using the Python matplotlib and seaborn library.

## Supplementary Information

Supplementary Information.

## Data Availability

All data necessary for interpreting the manuscript have been included.

## Code availability

Code is available upon request with a non-commercial license.
