# Supplementary material for: Closed-loop wearable naloxone injector system
Source: Sci Rep. 2021 Nov 22;11:22663. doi: 10.1038/s41598-021-01990-0 (PMC8608837; doi:10.1038/s41598-021-01990-0)
Supplement: Supplementary file 1 — Supplementary Information. [file 41598_2021_1990_MOESM1_ESM.pdf]

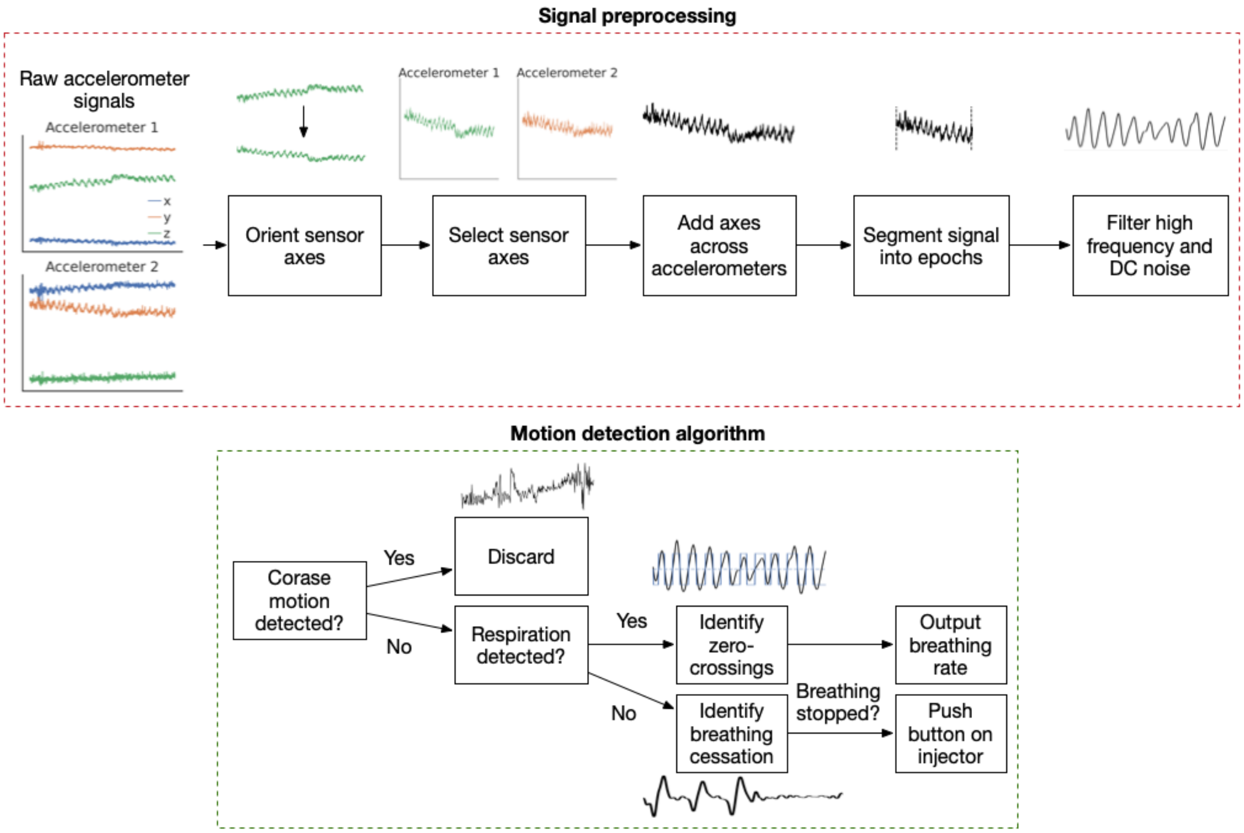

Supplementary Figure 1: **Pipeline of system operation.**

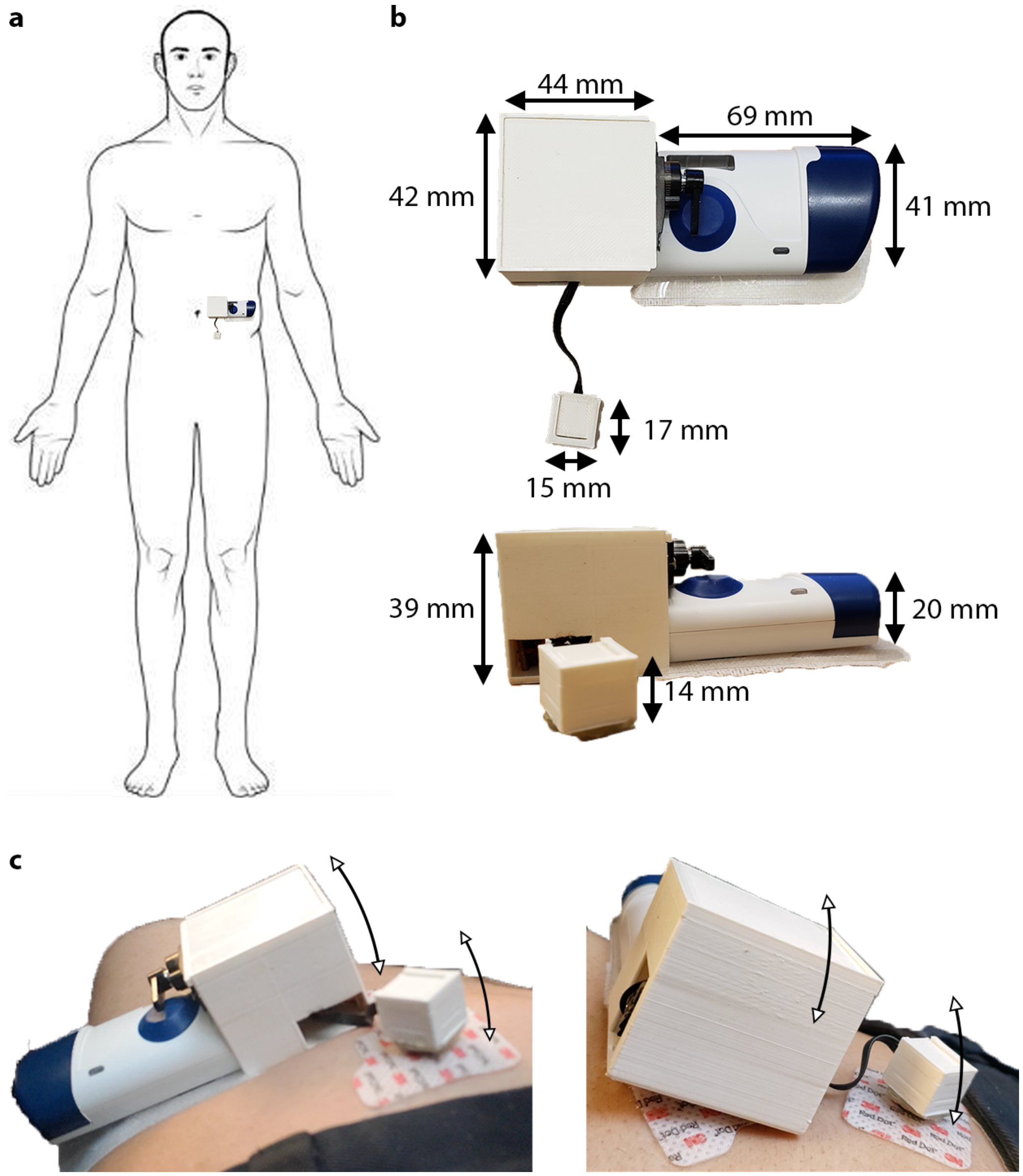

Supplementary Figure 2: **a,** Illustration of device position on body (Image source: https://www.template.net/design-templates/print/free-body-diagram/). **b,** Dimensions of device **c,** Different views of accelerometer movement during inhalation.

Algorithm 1: Algorithm to identify breathing cessation

Function `detect_breathing_cessation`($[x1, y1, z1, x2, y2, z2]$ $breathing\_counter, alert\_counter, mean\_signal$) **:**

  Input : $[x1, y1, z1, x2, y2, z2]$ accelerometer signals
  Input : $breathing\_counter$ Measure of how much of the signal is breathing
  Input : $alert\_counter$ Measure of how much of the signal is absent of breathing
  Input : $mean\_signal$ weighted average of signal amplitude over time
  $stop\_counter \leftarrow 0$
  for $axis \in [x1, y1, z1, x2, y2, z2]$ **do**
    $breathing\_signal \leftarrow bandpass(axis, [0.1, 1])$
    for $i \in breathing\_signal$ **do**
      if $i < mean\_signal[axis]$ *and* $i > -mean\_signal[axis]$ **then**
        $alert\_counter[axis]$ ++
        $breathing\_counter[axis] \leftarrow 0$
      else
        $breathing\_counter[axis]$ ++
      if $breathing\_counter[axis] > 5$ **then**
        $alert\_counter[axis] \leftarrow 0$
      if $alert\_counter[axis] \geq 300$ **then**
        $stop\_counter$ ++

    if $stop\_counter \geq 3$ **then**
      Activate injector

    $temp\_mean\_signal \leftarrow \frac{\sum breathing\_signal}{len(breathing\_signal)}$

    // update $mean\_signal$
    $mean\_signal[axis] \leftarrow 0.8 \cdot mean\_signal[axis] + 0.2 \cdot temp\_mean\_signal$

Algorithm 2: Algorithm to combine accelerometer signals

Function `combine_signals`(*[x1,y1,z1,x2,y2,z2]*) **:**

  Input : $[x1, y1, z1, x2, y2, z2]$ signals from both 3-axes accelerometers

  Output: $combined\_signal$ Combined breathing signal

  $max\_signal\_strength1 \leftarrow 0$

  $max\_signal\_strength2 \leftarrow 0$

  $max\_signal1 \leftarrow []$

  $max\_signal2 \leftarrow []$

  for $axis \in [x1, y1, z1, x2, y2, z2]$ **do**

    // orienting sensor axis

    $[maxs, max\_prominence] \leftarrow find\_maximums(axis)$

    $[mins, min\_prominence] \leftarrow find\_minimums(axis)$

    if $\frac{\sum maxs}{len(maxs)} < \frac{\sum mins}{len(mins)}$ *and* $\frac{\sum max\_prominence}{len(max\_prominence)} < \frac{\sum min\_prominence}{len(min\_prominence)}$ **then**

      $axis \leftarrow -axis$

    // selecting sensor axes

    $signal\_strength \leftarrow |max(maxs) - min(mins)|$

    if $signal\_strength > 5$ **then**

      if $axis \in [x1, y1, z1]$ *and* $signal\_strength > max\_signal\_strength1$ **then**

        $max\_signal1 \leftarrow axis$

        $max\_signal\_strength1 \leftarrow signal\_strength$

      else if $axis \in [x2, y2, z2]$ *and* $signal\_strength > max\_signal\_strength2$ **then**

        $max\_signal2 \leftarrow axis$

        $max\_signal\_strength2 \leftarrow signal\_strength$

  // adding axes across accelerometers

  $combined\_signal \leftarrow max\_signal1 + max\_signal2$

Algorithm 3: Algorithm to compute breathing rate

```
Function get_breathing_rate(epoch):
    Input  : epoch 30-second accelerometer signal
    Output: breaths_in_epoch number of breaths in the epoch
    //minimum distance between zero crossings
    xthresh ← 6

    // minimum amplitude change between zero crossings
    ythresh ← 0.5

    epoch ← get_breathing_rate_helper(epoch, xthresh, ythresh)
    vars ← Calculate variance for each one second segment in epoch
    vsum ← ∑ |vars|
    vmax ← max(|vars|)

    if vmax < 50000 and vsum > 20000 then
        Motion epoch; ignore.
    else
        Breathing epoch; count zero crossings.
        z ← 0
        for i ∈ len(epoch) − 1 do
            if epoch[i] · epoch[i + 1] < 0 then
                z ++
        breaths_in_epoch ← z/2
3

Function get_breathing_rate_helper(epoch,xthresh,ythresh):
    Input  : epoch 30-second accelerometer signal
    Input  : xthresh minimum distance between zero-crossings
    Input  : ythresh minimum amplitude change between zero-crossings
    Output: breathing_signal signal where periods of inhalation are marked as 1 and
            periods of exhalation are marked as -1
    last ← 0
    sum ← 0
    for i ∈ [0, ..., len(epoch)] do
        // if there is a zero-crossing
        if epoch[i] · epoch[i − 1] < 0 then
            if i − last > xthresh and sum > ythresh then
                for j ∈ [last, ..., i − 1] do
                    breathing_signal[j] ← sum
            last ← i
            sum ← 0
        else
            sum += |epoch[i]|
    for i ∈ [0, ..., len(epoch)] do
        if epoch[i] > 0 then
            breathing_signal[i] ← 1
        else
            breathing_signal[i] ← −1
```
